# Topological quantum states of matter in iron-based superconductors: from concept to material realization

**DOI:** 10.1093/nsr/nwy142

**Published:** 2018-11-21

**Authors:** Ning Hao, Jiangping Hu

**Affiliations:** 1 Anhui Province Key Laboratory of Condensed Matter Physics at Extreme Conditions, High Magnetic Field Laboratory of Chinese Academy of Sciences, Hefei 230031, China; 2 Beijing National Laboratory for Condensed Matter Physics and Institute of Physics, Chinese Academy of Sciences, Beijing 100190, China; 3 CAS Center of Excellence in Topological Quantum Computation and Kavli Institute of Theoretical Sciences, University of Chinese Academy of Sciences, Beijing 100190, China; 4 Collaborative Innovation Center of Quantum Matter, Beijing 100871, China

**Keywords:** iron-based superconductor, topological insulator, topological superconductor, spin–orbit coupling

## Abstract

We review recent progress in the exploration of topological quantum states of matter in iron-based superconductors. In particular, we focus on the non-trivial topology existing in the band structures and superconducting states of iron’s 3d orbitals. The basic concepts, models, materials and experimental results are reviewed. The natural integration between topology and high-temperature superconductivity in iron-based superconductors provides great opportunities to study topological superconductivity and Majorana modes at high temperature.

## INTRODUCTION

Over the past decade, topology has become an essential ingredient in the classification of various types of materials, including insulators/semiconductors, semimetals and superconductors [1–3]. The physical consequence in a topological material is the existence of topologically protected surface states, which can be measured directly in transport, angle-resolved photoemission spectroscopy (ARPES), scanning tunneling microscopy (STM) and other experiments [1–3]. In particular, in a topological superconductor, there are surface bound states, Majorana modes, which can be used to realize topological quantum computing because of their topological protection and non-Abelian braiding statistics [4].

While naturally born topological superconductors are very rare, the realization of Majorana modes can be achieved in many artificial hybrid systems. Recently, a wealth of proposals for such experimental designs has been proposed, including the superconducting surface states of a topological insulator in proximity to conventional superconductors [5], quantum wires with strong spin–orbit coupling in proximity to conventional superconductors [6], semiconductor–superconductor heterostructures [7], spin-chains embedded in conventional superconductors [8], etc. However, these hybrid systems, in general, have two shortcomings. First, it is always difficult to manage the interface between two different structures. Second, in all these proposals, as the proximity effect requires a long superconducting coherent length, high-temperature superconductors, such as cuprates and iron-based superconductors, have never been candidates for those integration processes because of their extreme short coherent lengths and structural incompatibility. Thus, all the devices need to be operated at very low temperatures.

The above shortcomings can be overcome if we can find a high-temperature superconductor that hosts non-trivial topological band structures. Specifically, to differentiate them from topological superconductors as well as the above hybrid superconducting systems, we refer to this type of superconductors specifically as *connate* topological superconductors [9]. The connate topological superconductor can be viewed as an internal hybrid system that has conventional superconductivity in the bulk but topological superconductivity on the surface caused by the non-trivial topology on some part of the band structures [9,10]. Because of this intrinsic hybridization, the superconductor, in general, must be a multiple band electronic system. As iron-based high-temperature superconductors are known to be multi-orbital electronic systems, they become promising candidates.

During the past several years, starting from theoretical understanding, research into iron-based superconductors as connate topological superconductors has gradually materialized. The first theoretical study of non-trivial band topology was carried out by us for the single-layer FeSe/STO, in which a band inversion can take place at the M points [11] to create non-trivial topology. Very quickly, it was found that the band inversion can easily take place at the Γ point if the anion height from the Fe layers is high enough. For FeSe, the height can be increased by substituting Se with Te [12,13]. For iron pnictides, the As height is predicted to be high enough in the 111 series, LiFeAs, to host the non-trivial topology [14]. Besides these intrinsic topological properties from the Fe d orbitals, non-trivial topology can also stem from bands outside the Fe layers. For example, the As p orbitals in the As layers of 122 CaFeAs_2_ are shown to be described by a model similar to the Kane–Mele model in graphene [15]. Most recently, because of the improvement of sample quality and experimental resolution, there has been increasing experimental evidence for topological properties in iron-based superconductors [16–18]. The theoretically predicted band inversions, together with the topologically protected surface states, have been directly observed. Majorana-like modes have been observed in several iron-chalcogenide materials [17,18]. All of this progress has made iron-based superconductors a new research frontier for topological superconductivity.

In this paper, we give a brief review of both the theoretical and experimental results regarding the topological properties of iron-based superconductors. In the section entitled ‘Topology in iron d-orbital bands’, we discuss theoretical concepts and models for the topological band structure in iron-based superconductors and recent experimental evidence. In the section entitled ‘Connate topological superconductivity’, we review the topological superconductivity that can emerge from the topological bands of iron-based superconductors and experimental evidence of Majorana-like modes in these materials. Finally, we will address open issues in this field.

## TOPOLOGY IN IRON D-ORBITAL BANDS

### Concepts and models

**Figure 1.**
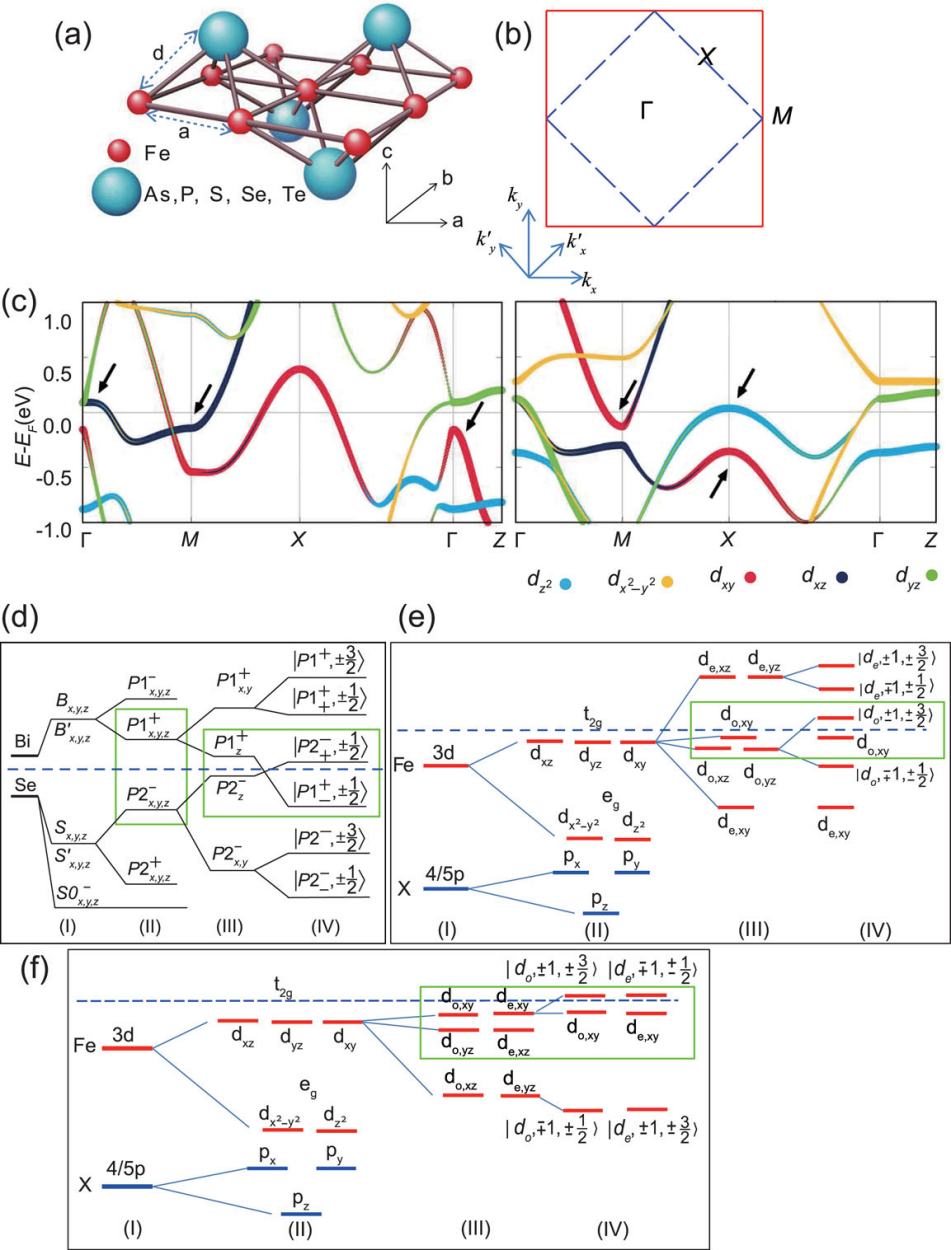
(a) The structure of the X–Fe–X trilayer. The distance between the two nearest-neighboring ions/ion and X is labeled with a and d. (Adapted from [23].) (b) The Brillouin zone with a high-symmetry point. The red solid/blue dashed lines label the Brillouin zone with a one-iron/two-iron unit cell. In (c) the top/bottom panels correspond to parameters (a, d) = (0.93,0.98)/(1.09,1) in units of experimental values of FeSe [20]. (d) Schematic picture of the origin of the band structure of Bi_2_Se_3_. Starting from the atomic orbitals of Bi and Se, the following four steps are required to understand the band structure: (I) the hybridization of Bi orbitals and Se orbitals, (II) the formation of the bonding and antibonding states due to the inversion symmetry, (III) the crystal field splitting, and (IV) the influence of the spin–orbit coupling, from [24]. (e) and (f) Similar processes in iron-based superconductors at the high-symmetry point Γ in (e) and point *M* in (f), from [25]. In both (e) and (f), (I) is the hybridization of iron 3d orbitals and X 4p or 5p orbitals, (II) the crystal field splitting, (III) the formation of the bonding and antibonding states, which are classified with the parities of glide-plane symmetry, and (IV) the influence of the spin–orbit coupling or other effects.

Since the discovery of iron-based superconductors in 2008, there has been remarkable progress in material growth and synthesis of the iron-based compounds. According to the element composition, iron-based superconductors are classified into different categories denoted by ‘1111’, ‘122’, ‘111’, ‘11’, etc. [19]. All categories possess the kernel substructure of an X–Fe–X trilayer with X denoting As, P, S, Se, Te, as shown in Fig. 1a. The X–Fe–X trilayer is the basic unit cell giving rise to magnetism and superconductivity, and plays a similar role to the Cu–O plane in cuprates. Following the principle from complexity to simplicity, the X–Fe–X trilayer skips the specificity among all the compounds in iron-based superconductors and brings the intrinsic physics to the surface. However, along the opposite logic, the diversity may include important subtle surprising differences. For iron-based superconductors, such kinds of accidental surprises can be intuitively demonstrated by evaluating the sensitivity of the electronic structures upon tiny changes in the structure of the X–Fe–X trilayer [20]. Figure 1c gives such an intuitive demonstration. The band structures sensitively depend on the fine-tuning of the distances between Fe–Fe and Fe–X. In particular, the bands switch orders near the Γ point, a band gap opens near the *M* point and the bands become strongly dispersive along the Γ–*Z* direction when the third dimension is considered. Indeed, the layered structures of the iron-based superconductors provide possibilities to tune the distances between Fe–Fe and Fe–X. For example, the La–O layer in LaOFeAs and the Ba–As layer in BaFe_2_As_2_ naturally cause different lattice constants for Fe–X layers [21,22]. A variety of materials in the family of iron-based superconductors provide different finely tuned X–Fe–X trilayers.

The band fine-tuning would become non-trivial if there exists a topological phase transition. The discovery of topological insulators has established a standard paradigm about the topological quantum states of matter, which includes band inversion, bulk–boundary correspondence, the relationship between symmetry and topological invariance, etc. [1,2,26–33]. For example, the first experimentally confirmed 2D topological insulator, the HgTe/CdTe quantum well, has a band inversion induced by the large spin–orbit coupling from Hg, depending on the thickness of the well, to give rise to a topological insulating state [27,28]. The well known 3D topological insulators Bi_2_Se_3_ and Bi_2_Te_3_ have band inversions caused by strong spin–orbit coupling that switches two p_$z$_-type bands with opposite spatial-inversion-symmetry parities at the Γ point [33–35]. The picture of band inversion can be further simplified into an energy-level shift in the atomic limit through adiabatic deformations [34]. Figure 1d gives a typical picture of the energy-level shift under the influence of several kinds of interactions in Bi_2_Se_3_ [24].

Interestingly, a similar picture also exists in some specific iron-based superconductors with finely tuned X–Fe–X layers. Typical pictures of the energy-level shifts of iron d orbitals are shown in Fig. 1e and f for the Γ point in (e) and the *M* point in (f), respectively. Note that the space group of the Fe–X–Fe trilayer is P4/*nmm*, in which the glide-plane mirror symmetry operation {$m_{z}|\frac{1}{2}\frac{1}{2}0$} and inversion symmetry operation {$i|\frac{1}{2}\frac{1}{2}0$} are essential [11,36–38]. First, the Bloch states can be classified according to the parities of {$m_{z}|\frac{1}{2}\frac{1}{2}0$}, i.e. |*d*_*o*/*e*, α_〉 or |*d*_*o*/*e*_, *m*_*l*_, *m*_*j*_〉 with *o*, *e*, α, *m*_*l*_, *m*_*j*_ denoting the odd or even parity of {$m_{z}|\frac{1}{2}\frac{1}{2}0$}, the αth d orbital, and two magnetic quantum numbers, respectively. Second, under the inversion symmetry operation {$i|\frac{1}{2}\frac{1}{2}0$}, the inversion parities of |*d*_*o*/*e*, α_〉 and |*d*_*o*/*e*_, *m*_*l*_, *m*_*j*_〉 for α = *xz*/*yz*, *m*_*l*_ = ±1 are opposite to the inversion parities of |*d*_*o*/*e*, α_〉 for α = *xy*. Focusing on the green rectangles in Fig. 1e and f, the spin–orbit coupling can switch the order of the energy levels with opposite inversion parities and induce a topological phase transition [11,37,38].

In early 2014, the authors of this paper noted a tiny band gap around the *M* point in the band structure of monolayer FeSe/SrTiO_3_ (FeSe/STO) [39] from ARPES measurement [40–45] and predicted the topological phase transition in this 2D system [11]. This is the first proposal discussing the topological quantum state of matter in iron-based superconductors. Corresponding to Fig. 1f, an effective **k** · **p** model can be constructed in the basis set of [{|ψ_*o*_〉}, {|ψ_*e*_〉}] with $\lbrace |\psi _{o}\rangle \rbrace =\lbrace |d_{o,xy,\uparrow }\rangle ,|d_{o},1,\frac{3}{2}\rangle ,|d_{o,xy,\downarrow }\rangle ,|d_{o},-1,-\frac{3}{2}\rangle \rbrace$ and $\lbrace |\psi _{o}\rangle \rbrace =\lbrace |d_{e,xy,\uparrow }\rangle ,|d_{e},-1,\frac{1}{2}\rangle ,|d_{e,xy,\downarrow }\rangle ,|d_{e},1,\\-\frac{1}{2}\rangle \rbrace$:

(1) \begin{equation*} H_{M}(k)=\left[ \begin{array}{c@{\quad}c} H_{M}^{o}(k) & H_{c}\\ H_{c} & H_{M}^{e}(k) \end{array} \right] . \end{equation*}

Here,

(2) \begin{equation*} H_{M}^{o}(k)=\left[ \begin{array}{c@{\quad}c} H(k) & 0\\ 0 & H^{\ast }(-k) \end{array} \right] , \end{equation*}

$H_{M}^{e}(k)=$$H_{M}^{o,\ast }(-k)$, *H*(*k*) = ε(*k*) + *d*_*i*_(*k*)σ_*i*_ with $\varepsilon (k)=C-D(k_{x}^{2}+k_{y}^{2})$, *d*_1_(*k*) + *id*_2_(*k*) = *A*(*k*_*x*_ + *ik*_*y*_), and $d_{3}(k)=M-B(k_{x}^{2}+k_{y}^{2})$ with *MB* > 0. In the absence of the *H*_*c*_ term, the Hamiltonian in Eq. (1) reduces into two copies of the Bernevig–Hughes–Zhang (BHZ) model [27], which is the standard model for the quantum spin Hall effect. In each subspace with odd or even parity, a topologically invariant *Z*_2_ = 1 can be defined. Actually, the *H*_*c*_ term is from the spin-flipped term λ_*so*_(*L*_*x*_*s*_*x*_ + *L*_*y*_*s*_*y*_), which mixes the orbitals with odd and even parities of {$m_{z}|\frac{1}{2}\frac{1}{2}0$}. As a consequence, the parity of {$m_{z}|\frac{1}{2}\frac{1}{2}0$} is no longer a good quantum number. The two subspaces couple with each other. The topological states are more of a weak type. However, if the two iron sublattices have different on-site potentials, i.e. the staggered sublattice potential, which is introduced by the substrate, the weak topological state can be tuned into strong topological states, because the potential can renormalize the mass term *M* in *d*_3_(*k*), and change its sign in only one copy. Now, the band inversion condition with *MB* > 0 is satisfied only in another copy. The topological state becomes strong and is robust against the *H*_*c*_ coupling without breaking time-reversal symmetry [11].

In late 2014, the topological phase transition around the Γ point was proposed in Fe(Te_1 − *x*_Se_*x*_) thin film [12], as well as in the bulk materials [13]. The first-principles calculations indicated that the proper ratio between Te and Se could induce the band inversion around the Γ point. Referring to Fig. 1e, an effective **k** · **p** model can be constructed in the basis set $\lbrace |d_{o},1,\frac{3}{2}\rangle ,|d_{o,xy,\uparrow }\rangle ,|d_{o},-1,-\frac{3}{2}\rangle ,|d_{o,xy,\downarrow }\rangle \rbrace$:

(3) \begin{eqnarray*} {H_{\Gamma }(k)=\varepsilon _{0}}\nonumber\\ &&\quad\quad\quad+\left[ \begin{array}{c@{\,\,}c@{\,\,}c@{\,\,}c} -M(k) & Ak_{+} & & \\ Ak_{-} & M(k) & & \\ & & -M(k) & -Ak_{-}\\ & & -Ak_{+} & M(k) \end{array} \right] .\nonumber\\ \end{eqnarray*}

Here, $\varepsilon _{0}=C-D(k_{x}^{2}+k_{y}^{2})$, $M(k)=M-B(k_{x}^{2} +k_{y}^{2})$. In the band inversion regime, *MB* > 0. Likewise, the effective **k** · **p** model around the Γ point in Eq. (3) restores the famous BHZ model that describes the quantum spin Hall effect in the HgTe/CdTe quantum well. In the original paper [12], the author considered hybridization between the p orbitals of Te/Se with the d orbitals of Fe. The basis functions for the **k** · **p** model would be complex. Here, we use only the d orbitals of Fe to construct the basis functions by downfolding the p-orbital parts without changing the symmetries. Therefore, the effective **k** · **p** models in the basis sets involving d and p orbitals or only d orbitals have identical forms.

**Figure 2.**
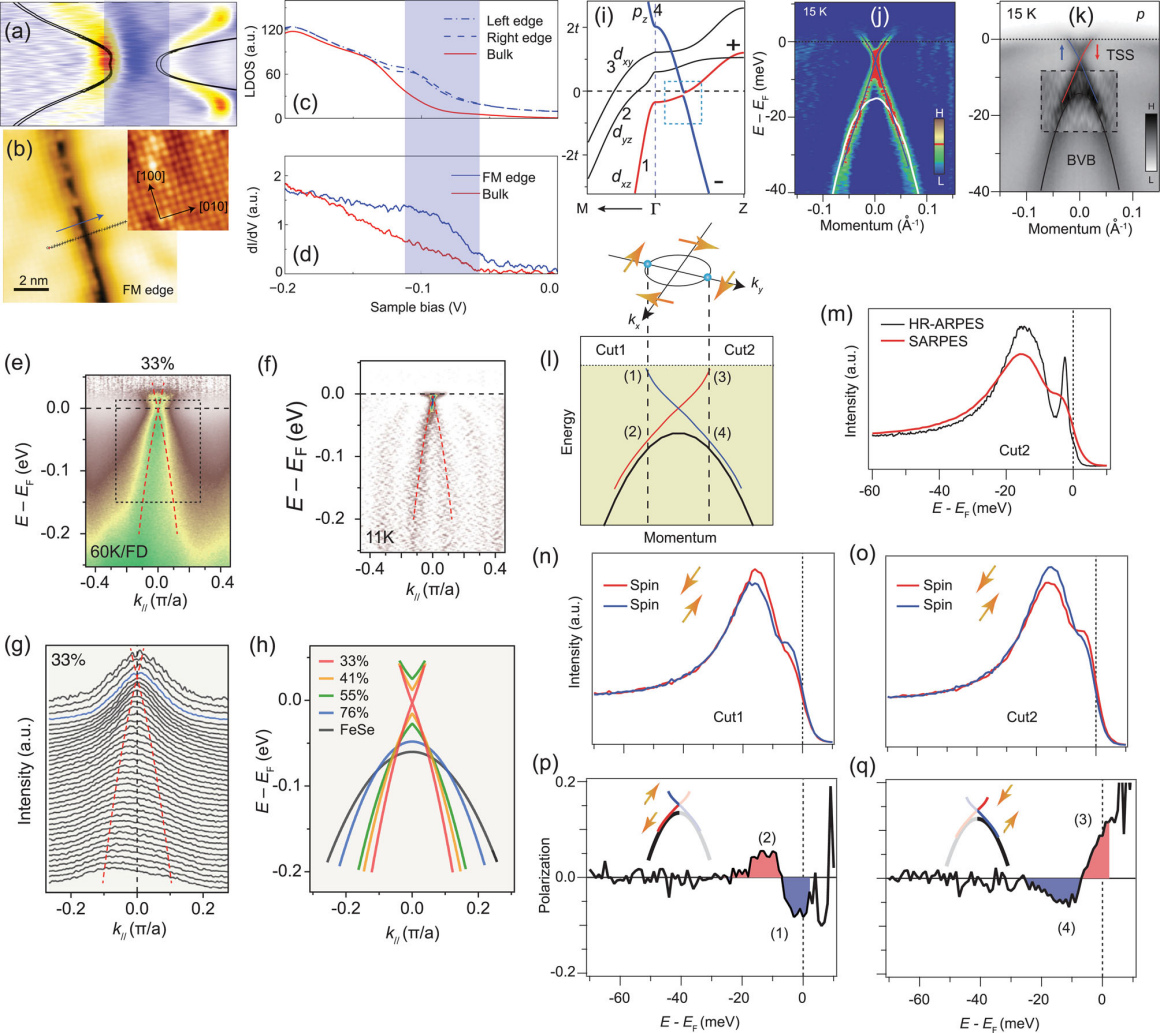
(a)–(d) The ARPES and STM experimental results for monolayer FeSe/STO [47]. (e), (f) The ARPES experimental results for monolayer Fe(Te_1 − *x*_Se_*x*_)/STO [9]. (i)–(q) The ARPES experimental results for bulk Fe(Te,Se) single crystal [16]. (a) ARPES band structure around the *M* point. The black lines are theoretical band structures. (b) Experimental STM topography of the FM edge (0.1 nA, −300 mV) of FeSe/STO. The inset shows an atomic-resolution STM topography image at the bulk position of the FM edge (0.1 nA, 100 mV), showing the topmost Se atom arrangement (the crystal orientations are labeled). (c) Theoretical local density of states (LDOS) for edge and bulk states. (d) Experimental STS spectra of edge and bulk states extracted from FM edges. The light blue band in (a), (c), (d) indicates the SOC gap. (e) The intensity plot divided by the Fermi–Dirac distribution function near Γ along the Γ–*M* direction for monolayer Fe(Te_1 − *x*_Se_*x*_)/STO. (f) Curvature intensity plots along the same cut as in (e). The data were recorded at the temperature indicated in the panel. (g) MDC plot corresponding to the spectrum in the black square in (e). (h) Comparison of the band dispersions at Γ for samples with different *x*. (i) First-principles calculations of band structure along Γ–*M* and Γ–*Z*. The dashed box shows the SOC gap of the inverted bands. (j) MDC curvature plot of the band data from ARPES, which enhances vertical bands (or the vertical part of one band) but suppresses horizontal bands (or the horizontal part of one band).The red dots trace the points where the intensity of the MDC curvature exceeds the red bar in the color-scale indicator, and the blue lines are guides to the eye indicating the band dispersion. (k) Summary of the overall band structure.The background image is a mix of raw intensity and EDC curvature (the area in the dashed box). The bottom hole-like band is the bulk valence band, whereas the Dirac-cone-type band is the surface band. (l) Sketch of the spin-helical FS and the band structure along *k*_*y*_, the sample Γ*M* direction. The EDCs at cuts 1 and 2 were measured with SARPES. The spin pattern comes from the bottom surface. (m) Comparison of the EDCs from SARPES and HR-ARPES measurements. The large broadening in the SARPES measurement could be partly responsible for the small spin polarization. (n) Spin-resolved EDCs at cut 1. (p) Spin polarization curve at cut 1. (o), (q) Same as (n) and (p), but for EDCs at cut 2. The measured spin polarizations are consistent with the spin-helical texture illustrated in (l).

The topological phase transition around the Γ point in the Fe(Te_1 − *x*_Se_*x*_) thin film can be generalized into the bulk Fe(Te_1 − *x*_Se_*x*_) single crystal. Correspondingly, the 2D topological state is generalized into 3D topological states, similar to the topological insulator in Bi_2_Se_3_. The topological nature of the band structures of bulk Fe(Te_1 − *x*_Se_*x*_) single crystal was proposed through the first-principles calculations [13]. The band inversion and *Z*_2_ topological invariance was revealed. Following the picture of topological phase transition at the Γ point shown in Fig. 1e, the topological phase transition in bulk Fe(Te_1 − *x*_Se_*x*_) single crystal is a little different from that in FeTe_1 − *x*_Se_*x*_ thin film. The spin–orbit coupling in the latter case does not play a primary role in the topological phase transition [12]. The spin–orbit coupling, however, is indispensable in the former case, because the small band gap between $\Gamma _{6}^{+}$ and Λ_6_ between the Γ–*Z* points is from the ‘transmission effect’, which transmits the coupling between $\Gamma _{4}^{+}$ and $\Gamma _{5}^{+}$ to the coupling between $\Gamma _{6}^{+}$ and Λ_6_ through the medium of spin–orbit coupling (see [13] for the relevant band labeling). The ‘transmission effect’ can be revealed by a tight-binding model only involving the five d orbitals of iron (the weight of the $|p_{z},\mathbf {k}+\mathbf {Q}\rangle$ state in the $\Gamma _{2}^{-}$ band can be renormalized to the $|d_{xy},\mathbf {k}\rangle$ state). The interlayer couplings include parity-conserved terms and parity-mixing terms [11]. Note that the $\Gamma _{2}^{-}$ state in the first-principles calculations is captured by band 4 in Fig. 2i. Without the interlayer parity-mixing term, even the spin–orbit coupling cannot open a gap between band 4 and bands 1, 2. Only when both the interlayer parity-mixing term and spin–orbit coupling are tuned on does a small band gap open, as shown in Fig. 2i. The key interlayer parity-mixing term is the hopping between *d*_*xz*_ and *d*_*yz*_, i.e. $-4it_{xz,yz}^{c}(\cos k_{x}+\cos k_{y})\sin k_{z}$. The effect of the interlayer parity-mixing term can be renormalized to obtain an effective spin–orbit coupling under the second-order perturbation approximation:

(4) \begin{equation*} \tilde{H}_{\mathrm{soc}}=\left[ \begin{array}{c@{\quad}c} 0 & \tilde{h}_{\mathrm{soc}}\\ \tilde{h}_{\mathrm{soc}}^{\dagger } & 0 \end{array} \right] , \end{equation*}

(5) \begin{equation*} \tilde{h}_{\mathrm{soc}}\propto \lambda _{\mathrm{soc}}[H_{c}^{\dagger }L^{-}+L^{-}H_{c}]. \end{equation*}

Here, λ_soc_ is the strength of the spin–orbit coupling. *L*^−^ is the matrix of d orbitals. *H*_*c*_ is the interlayer parity-mixing term. Along the Γ–*Z* line, (*k*_*x*_, *k*_*y*_) = (0, 0), we have

(6) \begin{eqnarray*} {\tilde{h}_{\mathrm{soc}}\propto it_{xz,yz}^{c}\lambda _{\mathrm{soc}}\sin k_{z}}\nonumber\\ &&\times\left[ \begin{array}{c@{\quad}c@{\quad}c@{\quad}c@{\quad}c} 0 & 0 & -i & 1 & 0\\ 0 & 0 & -1 & -i & 0\\ -i & -1 & 0 & 0 & -\sqrt{3}\\ 1 & -i & 0 & 0 & \sqrt{3}i\\ 0 & 0 & -\sqrt{3} & \sqrt{3}i & 0 \end{array} \right] . \end{eqnarray*}

Based on the information from the tight-binding Hamiltonian, the effective **k** · **p** Hamiltonian around the Γ–*Z* line can be constructed under the basis spanned by the states |1〉, |2〉, |3〉, and |4〉 in Fig. 2i [10]. The detailed form of the effective **k** · **p** Hamiltonian can be constructed in the basis set [{|ψ_↑_〉}, {|ψ_↓_〉}] with $\lbrace |\psi _{\uparrow }\rangle \rbrace =\lbrace |d_{e,xy,\uparrow }\rangle ,\,\,\, |d_{o},1,\frac{3}{2}\rangle ,\,\,\, |d_{o},-1,-\frac{1}{2}\rangle ,\,\,\, |d_{o,xy,\uparrow }\rangle ,\rbrace $ and $\lbrace |\psi _{\downarrow }\rangle \rbrace =\lbrace |d_{e,xy,\downarrow }\rangle ,|d_{o},1,\frac{1}{2}\rangle ,|d_{o} ,-1,-\frac{3}{2}\rangle , |d_{o,xy,\downarrow }\rangle \rbrace $:

(7) \begin{eqnarray*} {H_{\Gamma Z}(k) =}\nonumber\\ &&\left[ \begin{array}{c@{\quad}c@{\quad}c@{\quad}c} M_{1}(k) & \gamma \sin k_{z}k_{-} & \gamma \sin k_{z}k_{+} & 0\\ & M_{2}(k) & \alpha k_{+}^{2}+\beta k_{-}^{2} & i\delta k_{-}\\ & & M_{2}(k) & i\delta k_{+}\\ & & & M_{4}(k) \end{array} \right] \nonumber \\ && \otimes I_{2\times 2} + H_{\Gamma Z}^{\rm soc}(k). \end{eqnarray*}

Here, the mass terms $M_{n}(k)=E_{n}+\frac{k_{\parallel }^{2}}{2m_{nx}} +t_{nz}(1-\cos k_{z})$ with *n* = 1, 2, 4. $H_{\Gamma Z}^\mathrm{soc}$ are some components of $\tilde{h}_{\mathrm{soc}}$ in Eq. (6) and have the following form: $H_{\Gamma Z}^\mathrm{soc}(k)\,=\,\,[h_{11},\,h_{12};\,h_{12}^{\ast },\,-h11]\,\,\, {\rm with}$$h_{11}\,=\,\frac{\lambda _{\mathrm{soc}}}{2}[(\sigma _{z}-1)\oplus (\sigma _{z}+1)]$, $h_{12}\,=\,\frac{\sqrt{2}\lambda _{\mathrm{soc}}}{2} [i\sigma _{x}-\sigma _{y},(1-\sigma _{z})k_{z};1+\sigma _{z},(i\sigma _{x}+\sigma _{y})k_{z}]$.

Note that bands 4 and 2 cross along the Γ–*Z* line without a gap opening in Fig. 2i. Actually, this can be called a topological Dirac semimetal state when the chemical potential is moved to the cross point. This state can also be described by the effective model in Eq. (7). The target materials include Fe(Te,Se) and Li(Fe,Co)As [14,46].

### Materials and experiments

The three typical materials to realize the aforementioned topological quantum states of matter described by the three effective **k** · **p** Hamiltonians are monolayer FeSe/STO, monolayer FeTe_1 − *x*_Se_*x*_/STO and FeTeSe single crystal. To experimentally identify these topological states, scanning tunneling microscopy/spectroscopy (STM/S) and ARPES are very powerful tools. STM/S is a real space surface measurement technique that measures the density of states as a function of position, and can be used to distinguish the edge states from bulk states [48,49]. ARPES is a momentum space measurement technique that can directly read out the band structure, and can be used to evaluate the band evolution. The experimental results from STM/S and ARPES for these three materials are summarized in Fig. 2.

For monolayer FeSe/STO, the idea is based on comparing the gap (band gap and superconducting gap) from *dI*/*dV* of STS with the energy distribution curve (EDC) of ARPES in Fig. 2a to determine the bulk gap. Then, the topological states possess edge states, which cross the bulk band gap and are different from the trivial normal chemical edge states [47]. The contribution to the density of states from the topological edge states can be extracted by comparing the STS spectra between the bulk regime and the edge regime. Figure 2c and d shows the theoretical and experimental results, respectively. The key experimental observations are shown in Fig. 2c, from which one can find that there exist some additional states from the edges after subtracting the contributions from the bulk background. However, this feature alone is not enough to prove the non-trivial characteristics of the edge states. The trivial edge states can also have similar *dI*/*dV* behaviors. In [47], the checkerboard antiferromagnetic order is assumed to exist to open a trivial gap around the *M* point in the monolayer FeSe/SrTO. In [11], the trivial band gap at the *M* point is natural by taking into account the tension from the SrTiO_3_ substrate. Furthermore, the coexistence of antiferromagnetic order and superconducting order is doubtful in monolayer FeSe/STO, because the gap from the antiferromagnetic order is about 50 meV, which should be easy to detect. For example, the gap should disappear above the antiferromagnetic transition temperature T_*N*_. Thus, the non-trivial characteristics of the edge states should be further tested by other experimental method such as spin-resolved STM or non-local transport [50].

In Fe(Te,Se) thin films, the topological phase transition appears when increasing the Te substitution of Se. Pictorial band evolution about Te substitution is a convincing evidence for the topological phase transition in Fe(Te,Se) thin film. Therefore, an ARPES experiment is the primary choice. Figure 2h summarizes the band dispersions at the Γ point for samples with different *x*. The experimental results show that a down-shifting electron-like band moves towards the hole-like band and the band gap between them decreases rapidly when the Se content remains shrinks. Eventually the bands touch each other at a Se concentration of approximately 33%, which is further revealed in the plots of the constant energy contours and momentum distribution curves, as shown in Fig. 2e–g. The touch point corresponds to the critical point of band inversion. The ARPES experimental results give indirect evidence of the topological band structure in monolayer FeTe_1 − *x*_Se_*x*_/STO [9].

For the bulk FeTe_1 − *x*_Se_*x*_ single crystal, the emergence of electron band 4 in Fig. 2i is the key ingredient to produce topological states when increasing the Te substitution of Se. The early ARPES experiment proved its existence through introducing electron doping with *in situ* K evaporation [13]. The new high energy and momentum resolution ARPES (HR-ARPES) (energy resolution ∼70 μeV) and the spin-resolved ARPES (SARPES) (energy resolution ∼1.7 meV) provide powerful tools to directly observe the topological surface states and their spin polarization. Figure 2j and k clearly demonstrates the topological surface states with Dirac-cone structure. Figure 2n–q identifies the helical spin structure of the topological surface states. The combination of the HR-ARPES and SARPES results directly proves the topological band structure in the bulk FeTe_1 − *x*_Se_*x*_ single crystal [16]. Recently, similar topological band structure has also been identified in Li(Fe,Co)As [14], which not only confirms theoretical predictions but also proves the generic existence of tunable topological states in iron-based superconductors.

## CONNATE TOPOLOGICAL SUPERCONDUCTIVITY

### Material proposals

**Figure 3.**
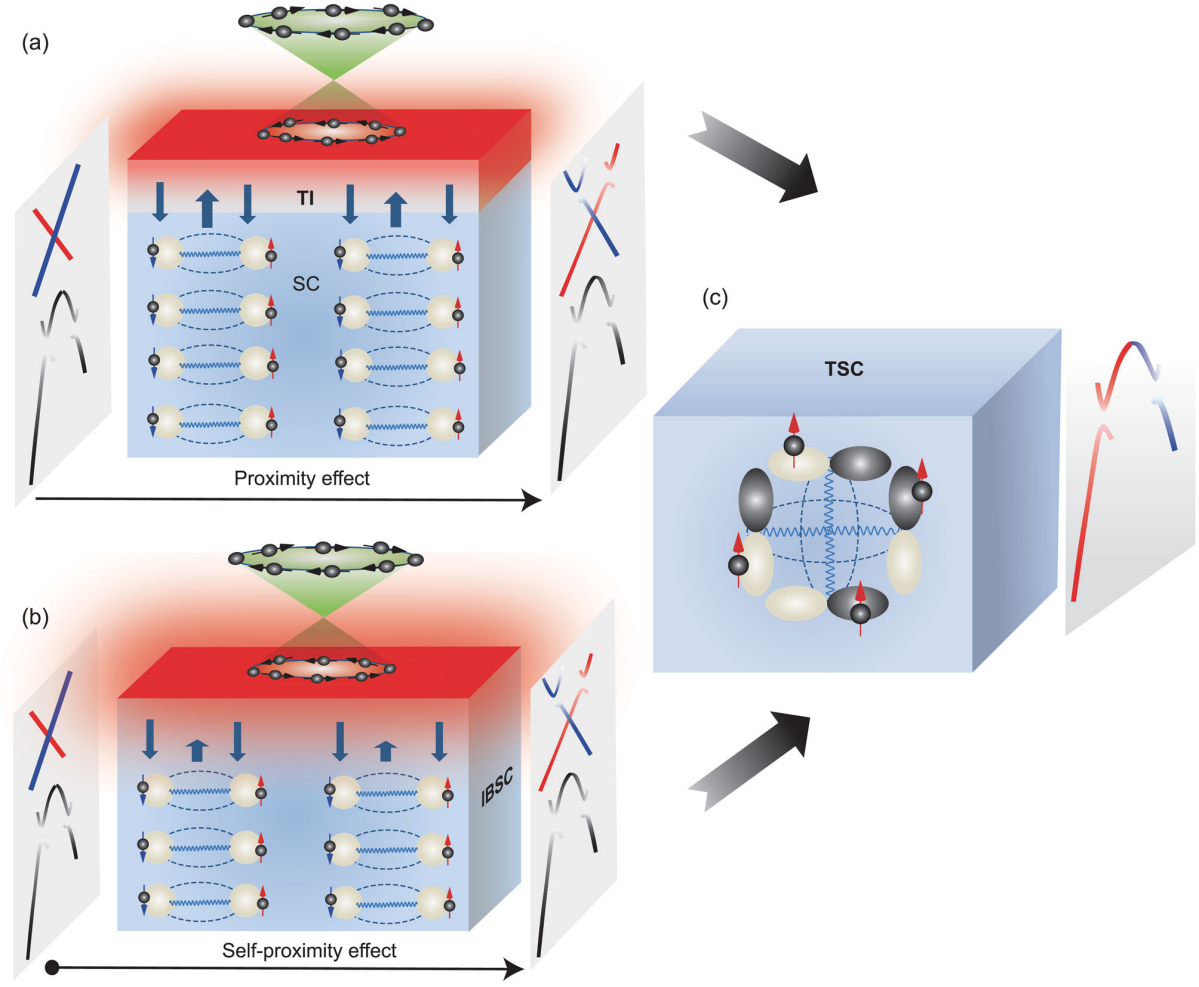
Schematic illustrations of three kinds of strategies to realize topological superconducting states. (a) Heterostructure involving a conventional s-wave superconductor and topological insulator film. (b) Iron-based superconductors with topological surface states. (c) Unconventional superconductors with odd-parity pairing, i.e. the spin-polarized *p* + *ip* pairing here.

As we mentioned in the introduction, a standard topological superconductor requires an odd-parity pairing, as shown in Fig. 3c. The famous representative materials, including Sr_2_RuO_4_ [51] and doped topological insulators Cu_*x*_Bi_2_Se_3_ and Sr_*x*_Bi_2_Se_3_ [52–59], are proposed as potential topological superconductors. However, the experimental situation is far from definitive, because the odd-parity pairing imposes restrictions on the pairing in the spin-triplet channel, which is very rare in solid-state materials. Therefore, recent research has mainly focused on some artificial structures that use the proximity effect from conventional superconductors on the surface/edge states of the 3/2D topological insulator, on semiconductor film/nanowire with strong Rashba spin–orbit coupling, and on iron atom chains [5–7,60–66], as shown in Fig. 3a. Effectively, the model describing the structure in Fig. 3a eventually reduces into the simpler model in Fig. 3c. The ultra-low superconducting transition temperature and the uncontrollability and uncertainty induced by the mismatch between different materials in the artificial structures cause many undetermined problems and take these structures far beyond practicality [65,66].

The superconductivity in iron-based superconductors is very robust against the fine-tuning of the band structures. Furthermore, the aforementioned topological phase transitions around Γ, *M* and the Γ–*Z* line have no overall band gap because the iron-based superconductors are of multi-orbital type and there exist other trivial bands across the Fermi energy besides the topological bands. When the temperature decreases below the superconducting transition temperature, the trivial bands across the Fermi energy open a superconducting gap due the formation of Cooper pairs. At the boundaries of the materials, the topological bands support the surface/edge states, which also cross the Fermi energy. In comparison with the trivial or extrinsic proximity effect involving two different kinds of materials in Fig. 3a, the inducing superconductivity from trivial bulk bands to topological boundary bands happens in a single material, and can also be called the intrinsic or self-proximity effect, as shown in Fig. 3b. When the Fermi energy is close to the surface Dirac point to guarantee a good approximation of the linear dispersion of the surface Dirac band, the superconducting single Dirac band can be reduced into a spinless *p*_*x*_ + *ip*_*y*_ superconductor [5,67], which is a topological superconductor, as shown in Fig. 3c. When the π-flux vortex is formed in the magnetic field, the effective topological superconductor can support zero-energy vortex-line end states, which are called Majorana modes.

Keeping the aforementioned picture in mind, one can find that all iron-based superconductors with topological band structures can support topological superconductors. For the monolayer FeSe/STO, the heavy hole-doped case can support topological edge states while the electron-doped case can support extremely high-temperature superconductivity. Then, the boundary between the hole-doped and electron-doped regimes in a single monolayer sample can produce a 1D topological superconductor. For monolayer FeTe_1 − *x*_Se_*x*_/STO, the superconductivity is robust in the whole doping regime [9]. The topological edge states emerge when *x* < 0.33, and the Cooper pairs from the electron bands near the *M* point can be scattered into topological edge states from topological bands near the Γ point. Then, the system spontaneously transforms into a topological superconductor. For (Ca,Pr)FeAs_2_ and Ca_1 − *x*_La_*x*_FeAs_2_, the distorted As chains in the CaAs layers support topological edge states through topological bands near the *B* points, while the FeAs layers support superconductivity through the trivial bulk band near both the Γ and *M* points. The self-proximity effect can induce 1D topological superconductivity in both (Ca,Pr)FeAs_2_ and Ca_1 − *x*_La_*x*_FeAs_2_ [15,68]. For bulk FeTe_1 − *x*_Se_*x*_ single crystal, the topological Dirac-cone-type surface states emerge at the $\bar{\Gamma }$ point in the (001) surface Brillouin zone in the topological doped regime. Then, the Cooper pairs from the trivial bulk bands near the Γ–*Z* line and the *M*–*A* line can be scattered into the topological Dirac-cone-type surface states. These primary and secondary self-proximity effects can drive the bulk FeTe_1 − *x*_Se_*x*_ single crystal into a 2D topological superconductor.

### Experiments and open questions

For the monolayer FeSe/STO and FeTe_1 − *x*_Se_*x*_/STO, the monolayer FeSe and FeTe_1 − *x*_Se_*x*_ grow on the substrate STO through the assistant of molecular beam epitaxy (MBE). So far, both systems have the highest superconducting transition temperature among all iron-based superconductors, whereas they are unstable in air. This shortcoming provides challenges for device fabrication and the relevant transport measurement. In contrast, the bulk FeTe_1 − *x*_Se_*x*_ single crystal is quite stable and has a nice (001) cleavage surface. More importantly, the topological superconducting states are 2D. The spontaneously generated vortex under external magnetic field could bound the Majorana zero-energy mode if the superconducting state is topological. Then, some experimental methods like ARPES and STM/S can be used to verify the topological superconducting state and detect the Majorana zero-energy modes. Based on these advantages, most experimental progress is mainly made in the bulk FeTe_1 − *x*_Se_*x*_ single crystal and (Li_0.84_Fe_0.16_)OHFeSe single crystal [16–18,69,70]. We review these experiments in chronological order below.

**Figure 4.**
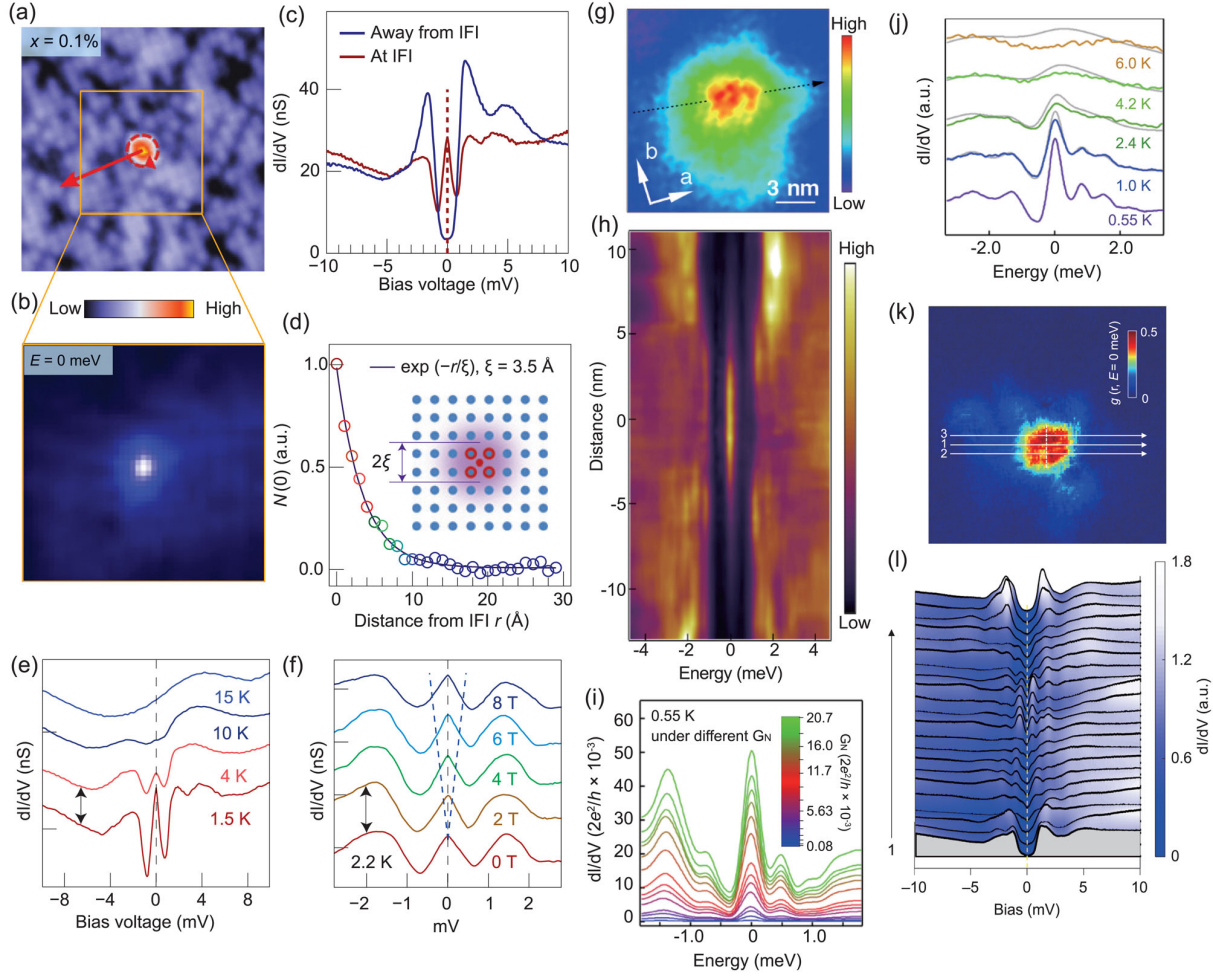
(a)–(f) The STM/S experimental results for zero-bias states trapped by interstitial Fe impurity in FeTe_0.57_Se_0.43_ [69]. (g)–(l) The STM/S experimental results for bound states trapped by the vortex in FeTe_0.55_Se_0.45_ [17,70]. (a) Topographic image of an isolated single interstitial Fe impurity (100 × 100 Å). (b) Zero-energy map for the area boxed in (a). (c) Spectra taken on top of and away from the interstitial Fe impurity. (d) Zero-energy peak value *N*(0) versus distance *r* from single interstitial Fe impurity. The solid curve is an exponential fit with ξ = 3.5 Å. Inset is a schematic image for the spatial distribution of interstitial Fe impurity scattering. (e) The spectra taken at the same interstitial Fe impurity at different temperatures. (f) The spectra taken at the same interstitial Fe impurity under different magnetic fields. The blue V-shaped dashed line is a guide to the eye showing the expected Zeeman splitting (*g* = 2). (g) A zero-bias conductance map (area 15 nm × 15 nm) around vortex cores. (h) A line-cut intensity plot along the black dashed line indicated in (g). (i) Evolution of zero-bias peaks with tunneling barrier measured at 0.55 K. *G*_*N*_ = *I*_*t*_/*V*_*s*_, which corresponds to the energy-averaged conductance of normal states, and represents the conductance of the tunneling barrier. *I*_*t*_ and *V*_*s*_ are the STS setpoint parameters. (j) Temperature evolution of zero-bias peaks in a vortex core. The gray curves are numerically broadened 0.55 K data at each temperature. (k) Image of a single vortex in a 20 nm × 20 nm region measured at 0.48 K and 4 T. (l) Tunneling spectra measured along the arrowed lines marked 1 in (k) with increment steps of 7.6 Å. The dashed line shows the position of the zero-bias voltage. The discrete CdGM bound-state peaks can be clearly observed near the vortex core center.

The first unexpected experiment is about the impurity bound states in FeTe_0.57_Se_0.43_ single crystals [69]. FeTe_0.57_Se_0.43_ single crystals contain a large amount of excess iron that as single iron atoms randomly situate at the interstitial sites between the two (Te, Se) atomic planes [71]. The STM/S spectrum observed a strong zero-energy bound state at the center of the single interstitial Fe impurity. The experimental results are summarized in Fig. 4a–f. The zero-energy bound state has the following features. (1) The spatial pattern of the zero-energy bound state is almost circular, which is different from the cross-shape pattern of the Zn impurity in Bi_2_Sr_2_Ca(Cu,Zn)_2_O_8 + δ_ [72]. (2) The intensity of the zero-energy bound state exponentially decays with a characteristic length of ξ = 3.5 Å, which is almost one order of magnitude smaller than the typical coherent length of 25 Å in the iron-based superconductor [73,74]. (3) The bound state is strictly at zero even when the external magnetic field increases to 8 T. (4) The zero-energy bound-state peak remains at zero energy even when two interstitial Fe impurity atoms are located near each other (∼15 Å).

It is a serious challenge to consistently explain these features of the zero-energy bound state induced by interstitial Fe impurity. The d-wave pairing symmetry scenario can result in a zero-energy bound state at the unitary limit [75], but this violates feature (1). The Kondo impurity resonance scenario can give an accidental zero-energy bound state [75], but this violates feature (4). A fascinating scenario is that the mode is a Majorana zero-energy mode [76,77], which captures features (1)–(3). Recently, a theoretical work claimed that an interstitial Fe impurity could bound an quantum anomalous vortex without a magnetic field, and the quantum anomalous vortex can bound a Majorana zero-energy mode when topological surface states of FeTe_0.57_Se_0.43_ become superconducting [78]. However, it is still hard to explain feature (4) by the Majorana zero-energy mode scenario. So far, the origin of the zero-energy bound state trapped by interstitial Fe impurity is still undetermined. Topological or other reasons need further experimental and theoretical exploration.

The second experimental breakthrough is about the vortex bound states on the surface of FeTe_0.55_Se_0.45_ single crystals [17,70]. FeTe_0.55_Se_0.45_ belongs to a type-II superconductor. Once a small external magnetic field is applied along the *c*-axis, magnetic vortex structures are formed due to the small lower critical field *H*_*c*1_. High-resolution STM/S can measure the bound states trapped by the vortex. Two experimental groups claimed completely different results for the same material, FeTe_0.55_Se_0.45_ single crystals. The former group claimed that they observed a sharp zero-bias peak inside a vortex core that does not split when moving away from the vortex center, which could be attributed to the nearly pure Majorana bound state [17]. The experimental results are summarized in Fig. 4g–j and l. The vortex bound states exhibit the following features. (1) Statistically, there is a success rate of about 20% in observing the isolated pure Majorana bound states during more than 150 measurements. (2) Across a large range of magnetic fields the observed zero-bias peak does not split when moving away from a vortex center. (3) Most of the observed zero-bias peaks vanish around 3 K. (4) Robust zero-bias peaks can be observed over two orders of magnitude in tunneling barrier conductance, with the width barely changing. Feature (1) is argued to be attributed to the disorder effect and/or inhomogeneous distribution of Te/Se. Feature (2) is attributed to the large Δ_sc_/*E*_F_ ratio in this system. Feature (3) is attributed to the idea that the Caroli–de Gennes–Matricon (CdGM) state [79] is protected by a mini-energy gap with a temperature of about $\Delta _\mathrm{sc}^{2}/E_\mathrm{F}\sim 3$ K, and thermal excitation around and beyond 3 K can kill the CdGM state. Feature (4) indicates that the line width of zero-bias peaks is almost completely limited by the combined broadening of energy resolution and STM thermal effect, suggesting that the intrinsic width of the Majorana bound state is much smaller in the weak tunneling regime [80,81]. The detailed experimental measurements eliminate some scenarios to cause a zero-bias peak in tunneling experiments, such as antilocalization, reflectionless tunneling, the Kondo effect, Josephson supercurrent and packed CdGM states near zero energy [56,82–88). Features (2)–(4) can be well understood with the Majorana bound-state scenario, so it is probable that the observed zero-bias peaks correspond to a Majorana bound state. However, feature (1) is a serious problem, which is different from other proposals to realize Majorana bound states. In the present experiments, it seems that no comprehensive evidence of the disorder effect and/or influence of the inhomogeneous distribution of Te/Se is provided. Furthermore, if the observed zero-bias peaks are from Majorana bound states, the non-Abelian statistics can be demonstrated by moving a vortex with an STM tip. This kind of experiment is the smoking gun for Majorana modes. Another experimental group claimed that they only observed the trivial CdGM bound state trapped by a vortex in the same FeTe_0.55_Se_0.45_ single crystals. For statistics, the energies of bound-state peaks close to the zero bias are collected from all nine measured vortices presented in [70]. The experimental results are summarized in Fig. 4k and l. In principle, there should be a special vortex to bound the zero-bias peak according to the 20% success rate claimed in the former experiment. Unfortunately, the two experiments for the same material from two groups give inconsistent results [17,70]. The argument attributing the difference to the different annealing processes is not very convincing. It seems that the appearance of zero-bias peaks is selective. The behaviors challenge the topological origin, which is usually universal and robust.

The third subsequent experiment is about the vortex bound states on the FeSe cleavage plane of (Li_0.84_Fe_0.16_)OHFeSe single crystal [18]. Compared with FeTe_0.55_Se_0.45_, the superconducting FeSe layers in (Li_0.84_Fe_0.16_)OHFeSe are stoichiometric. Therefore, there exist defect-free areas, which support the unpinned or free vortex idea. The STM/S measurements show that (1) the free vortex cores bound zero-bias modes, which do not shift with varying underlying superconducting gap as the other peaks do; (2) the zero-bias modes survive to high magnetic field due to the short coherence length; and (3) the zero-bias mode coexists with other low-lying CdGM states but they are separate from each other. These features are similar to those of the zero-bias modes observed in FeTe_0.55_Se_0.45_. Therefore, the zero-bias modes can also be attributed to Majorana zero-energy modes, and can be argued to have a topological origin in (Li_0.84_Fe_0.16_)OHFeSe. However, the topological origin in (Li_0.84_Fe_0.16_)OHFeSe is undetermined, unlike FeTe_0.55_Se_0.45_ with solid experimental evidence for the topological band structure. Recalling the discussions about the band inversion along the Γ–*Z* line in FeTe_0.55_Se_0.45_ in the section entitled ‘Concepts and models’, the strong dispersion of band 4 in Fig. 2j benefits from the quite small layer distance and the large size of Te atoms. Band 4 in pure FeSe is flat [13]. It is very strange that band 4 in (Li_0.84_Fe_0.16_)OHFeSe has strong dispersion. Furthermore, the band gap opening is due to the strong spin–orbit coupling from the Te atom, not the Se atom. Another critical condition to obtain the topological surface states is that the chemical potential must properly lie in the quite small band gap. However, the chemical potential in (Li_0.84_Fe_0.16_)OHFeSe is far from the band gap. In this situation, the top and bottom surfaces start to communicate with each other and break the zero-bias mode. Finally, it lacks the smoking-gun ARPES experiment to prove the helical structure of the claimed observed topological surface states in (Li_0.84_Fe_0.16_)OHFeSe. In summary, the experimental observations of the zero-bias modes in (Li_0.84_Fe_0.16_)OHFeSe are clearer, but the topological origin needs to be understood.

## SUMMARY AND PERSPECTIVES

The discovery of topological insulators has established a standard paradigm to guide the communities to pursue topological states of matter in quantum materials. Such pursuits cause intersections between the topology and iron-based superconductors. As emphasized in this review, important principles for the theoretical understanding of the energy-band topology in new materials include applying general concepts with the help of symmetry analysis and constructing effective models. For iron-based superconductors, the multi-orbital band structures and the diversity of materials provide opportunities to realize the effective theoretical models. These topological materials include monolayer FeSe/STO, monolayer Fe_1 − *x*_Se_*x*_/STO, FeTe_1 − *x*_Se_*x*_, LiFe_1 − *x*_Co_*x*_As, etc.

In the superconducting states, one naturally expects to obtain the topological superconducting states with the help of the self-proximity effect. However, unlike the energy-band topology, the expected topological superconducting states exhibit many unexpected experimental phenomena, including the surprising robust zero-energy mode trapped by Fe impurity in FeTe_0.57_Se_0.43_, the selective appearance of the zero-bias mode trapped by the vortex in FeTe_0.57_Se_0.43_, and the coexistence of the zero-bias mode and CdGM states trapped by the free vortex in (Li_0.84_Fe_0.16_)OHFeSe. Even if all these phenomena are attributed to Majorana zero-energy modes, there are deep inconsistencies within different experiments as well as between experiments and theories. In this respect, clarifying the creation mechanism of these so-called Majorana zero-energy modes is worth pursuing. For such efforts, the availability of high-quality single crystal, whose chemical potential can be artificially finely tuned, would be crucial. Once the physics of the so-called Majorana zero-energy modes is clarified, finding ways to manipulate the non-Abelian statistics of the Majorana zero-energy modes is a significant challenge for future applications in quantum computing.

Iron-based superconductors have rich phase diagrams. Beside the normal and superconducting phases, there are nematic, orbital ordering and various antiferromagnetic phases. Searching the topology embedded in these ordered phases would be interesting. For the theoretical aspect, there have been some studies [89,90], but the experimental exploration is blank. In future, a stronger collaboration between theory and experiment is required to explore topological quantum states in the new materials of iron-based superconductors.

Finally, it cannot be entirely ruled out that the superconducting states of iron-based superconductors themselves could be highly unconventional. Despite the 10 years of research into iron-based superconductors, there still are many unsolved puzzles [91,92] observed by a variety of different experimental methods, such as transport, Raman spectra, neutron scattering, nuclear magnetic resonance, electron spin resonance, STM/S, ARPES, etc. For example, the interplay between spin, orbital, lattice and charge degrees of freedom is not fully understood; not only is there no smoking-gun proof for the s_±_ pairing yet but it is also clear that the s_±_ pairing symmetry cannot be valid for many iron-chalcogenide systems; whether there is a sign change in the superconducting states of iron-chalcogenide systems without hole pockets or not is highly debated; and the origin of the enhancement of the transition temperature found in single-layer FeSe remains to be understood. The topological exploration in iron-based superconductors may help us to discover surprising characters and mechanisms hidden behind the superconducting pairing, and lead to answers to these unsolved puzzles.
